# Extending the Horizon of Homology Detection with Coevolution-based Structure Prediction

**DOI:** 10.1016/j.jmb.2021.167106

**Published:** 2021-10-01

**Authors:** Luis Sanchez-Pulido, Chris P. Ponting

**Affiliations:** Medical Research Council Human Genetics Unit, Institute of Genetics and Cancer, University of Edinburgh, Edinburgh EH4 2XU, UK

**Keywords:** remote homology, CFAP298, C21ORF59, DISC1, coevolution, BLAST, Basic Local Alignment Search Tool, C21ORF59, Chromosome 21 Open Reading Frame 59, CATH, Class Architecture Topology Homologous, CFAP298, Cilia- and flagella-associated protein 298, DISC1, Disrupted in schizophrenia 1, MON1A, Monensin sensitivity protein 1A, MSA, Multiple sequence alignment, PDB, Protein Data Bank, Pfam, Protein Families Database, STRING, Search Tool for the Retrieval of Interacting Genes/Proteins

## Abstract

Traditional sequence analysis algorithms fail to identify distant homologies when they lie beyond a detection horizon. In this review, we discuss how co-evolution-based contact and distance prediction methods are pushing back this homology detection horizon, thereby yielding new functional insights and experimentally testable hypotheses. Based on correlated substitutions, these methods divine three-dimensional constraints among amino acids in protein sequences that were previously devoid of all annotated domains and repeats. The new algorithms discern hidden structure in an otherwise featureless sequence landscape. Their revelatory impact promises to be as profound as the use, by archaeologists, of ground-penetrating radar to discern long-hidden, subterranean structures. As examples of this, we describe how triplicated structures reflecting longin domains in MON1A-like proteins, or UVR-like repeats in DISC1, emerge from their predicted contact and distance maps. These methods also help to resolve structures that do not conform to a “beads-on-a-string” model of protein domains. In one such example, we describe CFAP298 whose ubiquitin-like domain was previously challenging to perceive owing to a large sequence insertion within it. More generally, the new algorithms permit an easier appreciation of domain families and folds whose evolution involved structural insertion or rearrangement. As we exemplify with α1-antitrypsin, coevolution-based predicted contacts may also yield insights into protein dynamics and conformational change. This new combination of structure prediction (using innovative co-evolution based methods) and homology inference (using more traditional sequence analysis approaches) shows great promise for bringing into view a sea of evolutionary relationships that had hitherto lain far beyond the horizon of homology detection.

## Highlights

• Co-evolution-based methods extend the homology detection horizon of protein sequence analysis.
• Predicted contact maps help predict domain boundaries and tertiary structures.
• Such maps also aid in identifying internal repeats and large insertions.
• Coevolution-based methods could yield insights into protein dynamics and conformational change.

## Graphical abstract

**Figure:**
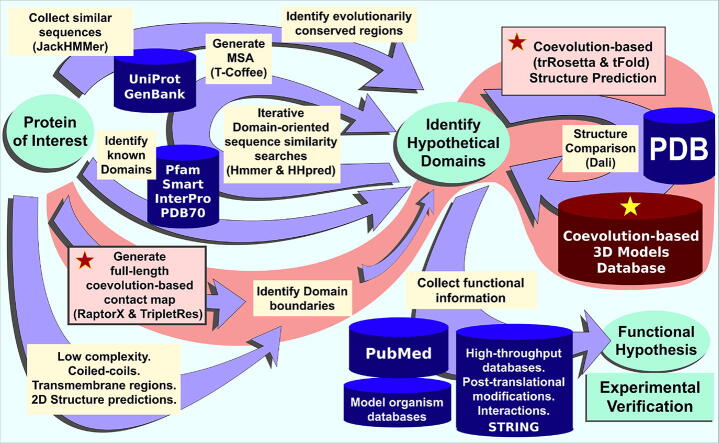

## Introduction

Homology is the most reliable basis by which to transfer functional information from experimentally investigated proteins to their more poorly understood homologues.^1^ Although investigators often find this ability to predict function invaluable, they can be frustrated when dealing with a protein that apparently lacks experimentally-investigated homologues. Even information from distantly-related homologues might aid their generation of experimentally-tractable hypotheses. Yet too often these homologues lie out of sight, beyond the detection horizon – the distance beyond which homology is not predictable.^2^

Features that are shared due to evolutionary descent from a common ancestor are homologous. For proteins, such features can span their full length or else be limited to a structural element such as a constituent domain or motif.^3^ When these sequences are closely similar, exceeding for example 40% amino acid identity over long alignments, they are then unambiguously homologous and thus within the detection horizon. With lower levels of identity, however, homologues are more distantly related, having accumulated sequence elaborations over millions or billions of years of evolution that left only functionally-required catalytic or binding site residues relatively unaltered. At this evolutionary distance separating a pair of sequences, the amino acid identity of homologues can be so low (~10%) as to be no different from pairwise identities of randomly sampled non-homologues,^4^ they then lie beyond the detection horizon.

Nevertheless, sometimes homologues and non-homologues can be distinguished just within the detection horizon. Algorithms such as PSI-BLAST^5^ and JackHmmer^6^ were developed to analyse not just a pair of sequences, but many, so as to extract additional evolutionary information inherent within their multiple sequence alignment (MSA).^7^ MSAs reflect variation in how far protein sequences can diverge whilst both conserving their molecular function and maintaining the overall arrangement and topological connections of their protein secondary structures. PSI-BLAST and JackHmmer build up these MSAs iteratively, searching sequence databases for ever more divergent homologues. Methods such as HHpred were subsequently devised to evaluate the similarity between two MSAs, allowing more remote evolutionary relationships to be discovered.^8^, ^9^ Careful application of such approaches can unambiguously assign proteins and their domains to very ancient families, even in the face of their meagre residual sequence similarity. As one of many examples, metazoan Treslin/Ticrr was identified as a homologue of yeast Sld3 despite only slight sequence similarity between them, thereby neatly explaining these molecules’ comparable roles in the pre-initiation step of eukaryotic DNA replication.^10^

These established database search algorithms have three major limitations. The most important of these is that they cannot detect distant homologues lying beyond the detection horizon.^4^ This horizon is determined by the evolutionary information within the MSA that is extracted by the algorithm. PSI-BLAST and JackHmmer extract this information by scoring conservation at each MSA position independently of every other position, without accounting for correlated evolutionary sequence changes.^5^, ^6^ The primary subject of this review article is how contact and distance map prediction can now extract additional evolutionary information from correlated sequence substitutions that further extends the horizon of homology detection.

These algorithms’ second limitation derives from proteins having evolved by the fusing and deletion of domains in diverse combinations. This creates a plethora of different domain architectures^11^, ^12^, ^13^ and substantial confusion when interpreting these algorithms’ sequence alignments. For example, algorithms can align sequences *A* and *B* to a query *Q* despite *A* and *B* not being homologous, specifically when *Q* contains two domains that are homologues of either *A* or *B* but not both. Optimally, homology should be detected for a single discrete evolutionary unit such as a domain.^3^ Researchers, however, if they are not prescient of their protein-of interest’s domain architecture, cannot disassemble it into its constituent domains to allow their subsequent querying by these algorithms. Querying proteins containing repeated domains or structures (“repeats”) using these algorithms often yields bewildering combinations of alignments to single protein sequences. Again, these algorithms’ use on such sequences optimally requires users to be forewarned of their repeat content.^14^

The final limitation relates less to these sequence database search algorithms, and more to how we interpret their results. Domains in proteins are usually arranged as “beads-on-a-string”.^15^ Exceptions to this more general rule include proteins in which one domain has been inserted within another.^16^, ^17^ These instances challenge our interpretation of alignments because commonly our assumption is that aligned sequence segments are collinear and lie in close spatial proximity. Domains that are not “beads-on-a-string” are often overlooked at present even when their sequence similarities are revealed by these algorithms.

In this review, we explain how recent advances in coevolution-based structure prediction now promise to push back the homology detection horizon yet further. The concept that is central to these new methods is their ability to predict three-dimensional structure, which is well-known to be better conserved than a protein’s primary sequence.^18^ With these methods now at hand, it is neither sequence nor structural similarities that correctly predicts protein homology: it is the combination of the two.

## What is Coevolution-based Contact and Distance Prediction?

A contact or distance map is a two-dimensional (2D) representation of a 3D protein structure representing a matrix of residue-to-residue distances.^19^ Where two secondary structures lie in close spatial proximity, for example a pair of adjacent α-helices, the map contains an internal feature that signifies multiple contacts between spatially proximal residues. The number and arrangement of features in a contact map are thus characteristic of the protein’s overall fold.^20^

**Figure 1:**
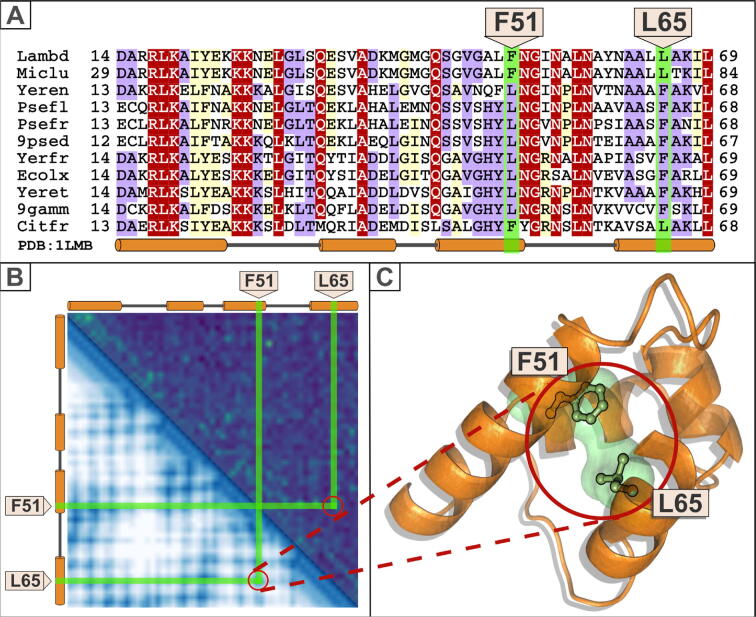
Coevolved positions. Correlated mutations between amino acids predict their proximity in a protein structure.^21^, ^22^, ^23^, ^24^, ^25^ In this example, taken from one of the earliest analysis of compensatory mutations,^31^ an intra-protein interaction is maintained via a hydrophobic (Phe-Leu) interaction in the core of the lambda repressor structure. An initial mutation at one of the two sites is disadvantageous but becomes fixed upon a compensatory mutation being introduced at a second site: substitutions are correlated between positions F51 and L65. In this scenario, the compensatory mutation is advantageous, and thus positively selected. The pair of substitutions, however, would not be adaptive unless together they enhance fitness.^31^, ^34^, ^35^ (A) Representative multiple sequence alignment (MSA) of the lambda repressor family. The alignment is presented using the program Belvu.^93^ Secondary structure of *Escherichia* phage lambda repressor is shown below the alignment (PDB: 1LMB_A).^94^ Alpha-helices are indicated by cylinders. The sequences are named with their UniProt species abbreviation: Lambd, P03034, *Escherichia* phage lambda; Miclu, D3LKV4, *Micrococcus luteus*; Yeren, F4MUG6, *Yersinia enterocolitica*; Psefl, A0A5E6YEE5, *Pseudomonas fluorescens*; Psefr, A0A6H3BJL2, *Pseudomonas fragi*; 9psed, I4KV84, *Pseudomonas synxantha*; Yerfr, A0A0B6FWC7, *Yersinia frederiksenii*; Ecolx, A0A5N9VVB6, *Escherichia coli*; Yeret, A0A3S6EWE6, *Yersinia entomophaga*; 9gamm, A0A2A2GYD8, *Arsenophonus sp.*; Citfr, A0A064E0Q9, *Citrobacter freundii*. (B) Above the diagonal is shown the Markov Random Fields (MRF) matrix (correlations) calculated from an extended version of the alignment shown in (A). Below the diagonal is shown a residue-residue distance matrix resulting from a subsequent deep learning analysis. Both matrices were generated using the tFold server (https://drug.ai.tencent.com/console/en/tfold) (C) Cartoon of the lambda repressor core structure of *Escherichia* phage lambda (PDB: 1LMB_A)^94^ generated using PyMOL (https://pymol.org/). F51 and L65 amino acid side chains are shown in sticks and are labelled.

Contact and distance maps have recently received greater attention owing to their use in predicting protein fold and structure from amino acid sequence.^21^, ^22^, ^23^, ^24^, ^25^, ^26^ For this purpose, rather than being calculated from structural data, these maps are predicted from MSAs (Figure 1). To understand how these maps are constructed we return to MSAs, and the assumption made by conventional sequence analysis algorithms (e.g. PSI-BLAST and JackHmmer) that each alignment position evolves independently of any other. This approximation ignores how MSA positions co-vary, due to substitutions occurring within them not being independent. Covariation of substitutions is not a minor effect. Rather, correlated amino acid substitutions are widespread with most neutral or beneficial substitutions found in one species being disadvantageous in others.^27^

Correlated mutations identified in a large MSA indicate a tendency for positions in proteins to mutate co-ordinately.^28^, ^29^ A major reason for such mutations is the maintenance of a protein’s tertiary structure,^30^, ^31^, ^32^, ^33^, ^34^, ^35^ for example to preserve a physical interaction between two distal portions of a protein’s sequence (Figure 1). When mutations are correlated it can thus be hypothesised that they lie in close spatial proximity. It is this inference of pairwise spatial constraints from correlated substitutions that is now being exploited to predict protein folds.^21^, ^22^, ^23^, ^24^, ^25^ The number of such spatial constraints necessary for predicting a fold can be surprisingly few once they are combined with other constraints, derived from the primary sequence and the geometry of protein secondary and tertiary structure.^36^

Various methods that take advantage of correlated mutations (Table 1) have been applied successfully to single globular domains, long protein sequences and transmembrane proteins.^21^, ^22^, ^23^, ^24^, ^25^, ^37^, ^38^, ^39^, ^40^, ^41^, ^42^, ^43^, ^44^, ^45^, ^46^ For further information on these methods we refer the reader to websites provided in Table 1, and to other contributions to this Special Issue.

**Table 1:** Selected coevolution-based methods in protein structure analysis

Name of method	Web-server	Reference
**Coevolution-based contact prediction**		
RaptorX	http://raptorx.uchicago.edu/ContactMap	22
TripletRes	https://zhanglab.ccmb.med.umich.edu/TripletRes	41
DeepMetaPSICOV	http://bioinf.cs.ucl.ac.uk/web_servers	42
**Coevolution-based protein structure prediction**		
trRosetta	https://yanglab.nankai.edu.cn/trRosetta	23
tFold	https://drug.ai.tencent.com/console/en/tfold	
C-QUARK	https://zhanglab.ccmb.med.umich.edu/C-QUARK	
MULTICOM	http://sysbio.rnet.missouri.edu/multicom_cluster	43
**Domain boundaries prediction**		
FUpred	https://zhanglab.ccmb.med.umich.edu/FUpred	44
**Structure prediction for Transmembrane Proteins**		
MemBrain	http://www.csbio.sjtu.edu.cn/bioinf/MemBrain	45
PureseqTM	http://pureseqtm.predmp.com	46

**Figure 2:**
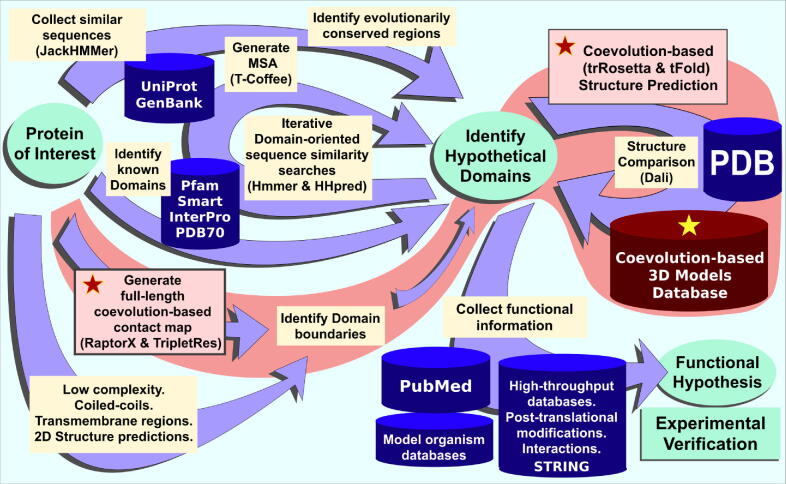
Schematic flowchart outlining analyses of protein sequence, structure, and function. A pair of pink rectangles included in the red work-flow (labelled with red stars) indicate two approaches now available thanks to coevolution-based contact predictions. In the first, full-length protein sequence coevolution-based contact maps (using RaptorX, TripletRes, or FUpred) help to delimit domains based on their high density of internal contacts.^22^, ^41^, ^44^ In the second, coevolution-based 3D structure models (generated with tFold or trRosetta, for example) can be compared against the Protein Data Bank (PDB)^57^, ^58^, ^95^, ^96^ and against large databases of coevolution-based 3D models (labelled with a yellow star) thereby allowing unexpected structural similarities to be revealed. Recently, a new set of coevolution-based 3D models has been generated using trRosetta for more than 6300 domains annotated in Pfam.^23^, ^85^ We typically initiate our analyses by collating homologues of the protein of interest using JackHMMER searches of the UniRef50 database.^6^, ^97^ We also check for annotated domains in our protein sequence using Pfam, Smart, InterPro and PDB70.^9^, ^85^, ^98^, ^99^ Subsequently, we construct MSAs (using, for example, T-Coffee)^100^ seeking to identify evolutionarily conserved regions which we then enrich with more remotely related sequences by iterative domain-oriented sequence similarity searches using Hmmer and HHpred.^6^, ^9^ Prior to commencing such an analysis, it is advisable to use different servers that predict diverse attributes in our protein of interest, such as low complexity, coiled-coils, transmembrane regions, and secondary structure predictions.^101^, ^102^, ^103^, ^104^ Domain boundaries predictions in proteins with unknown structure traditionally were based on evolutionarily conserved regions identified among different protein families, complemented by the prediction of interdomain linker regions.^48^, ^105^ Domain identification is a complex task, even if structures have been determined experimentally. For example, CATH, a database of protein domain structures, when identifying domains and their boundaries, uses the result of three different methods (PUU, DETECTIVE, and DOMAK) to guide manual annotation, each requiring a high degree of human specialization.^106^ A final step in our analysis is function prediction (bottom right). For this task we bear in mind that knowledge of a protein’s tertiary structure does not necessarily establish its molecular function. In recent years, structural genomics projects have deposited many structures in PDB without adding functional annotation, mainly due to their absence of sequence similarity with proteins of experimentally determined function.^1^, ^107^ Detailed consideration and analyses of the literature and high-throughput (including protein–protein interaction) data are required. A tool that aids in this task is STRING, which integrates known and predicted associations among proteins.^108^ Our analysis is considered successful if it leads to experimentally-tractable functional hypotheses, helping to gain mechanistic insight into our target protein under non-pathological and/or pathological conditions.

The availability of contact and distance prediction maps should now help push back the detection horizon of distant homology. Faced with an innocuous protein sequence apparently devoid of distinguishing features, a researcher can now infer the sequence boundaries of its structural building blocks – its domains and repeats – to ‘carve up’ the sequence into putative constituent domains or repeats, permitting these to be used piecemeal in individual sequence database searches (Figure 2).

### Domain boundaries

**Figure 3:**
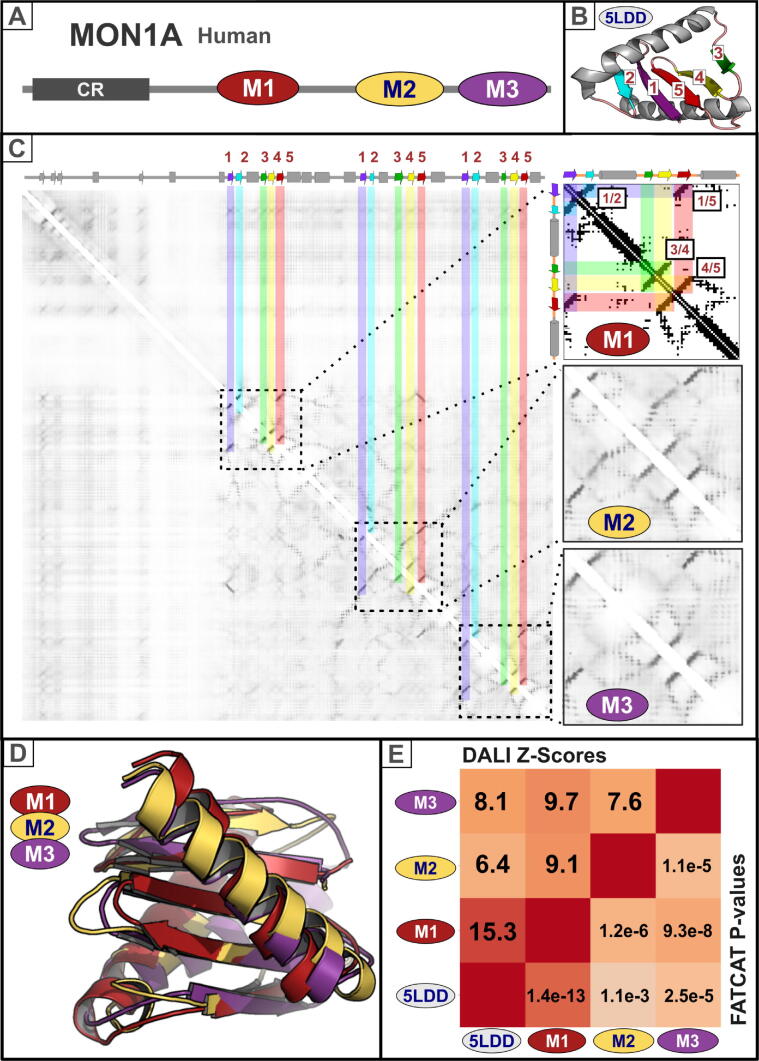
Identification of domain boundaries and internal domain duplications. Three Longin domains in MON1A. (A) Domain architecture of human MON1A protein (isoform 5, Uniprot: Q86VX9-5). The red oval (labelled M1) represents the first Longin domain in MON1A identified by sequence similarity analysis by Kinch and Grishin.^109^ The crystal structure of this first Longin domain was later solved in the fungus *Chaetomium thermophilum* MON1 protein.^53^ Using two contrasting evolutionary analyses, sequence conservation and coevolution-based contact predictions, we recently identified two additional Longin domains in MON1A (yellow and violet ovals labelled M2 and M3).^52^ Abbreviation: CR, conserved region. (B) Cartoon of the Longin domain structure of the *C. thermophilum* MON1 core structure (PDB: 5LDD_A; amino acids 222–316) generated using PyMOL (https://pymol.org/). Beta-strands are labelled 1 to 5 and coloured in purple, cyan, green, yellow, and red, respectively. https://pymol.org/(C) MON1A contact maps. Left: Coevolution-based contact map for full-length human MON1A protein predicted by RaptorX using default parameters.^22^ PsiPred secondary structure predictions^103^, ^104^ for MON1A are shown above the contact map. A triplicated 2D contact pattern is observed, labelled M1, M2 and M3 (right). For each MON1A Longin domain its β-strands 1 to 5 are coloured purple, cyan, green, yellow, and red, respectively. Right: (Top) The actual contact map calculated from the known structure of the first Longin domain (M1) of *C. thermophilum* MON1 (PDB: 5LDD_A), generated using the Cocomaps server (input: 5LDD_A *versus* 5LDD_A, cut-off distance value = 7 Å).^110^ Spatially adjacent anti-parallel β-strand pairs, located within a β-sheet, are labelled (β-strand pairs 1/2, 1/5, 3/4 and 4/5). Two similar Longin-like contact patterns, predicted with RaptorX,^22^ are observed in two consecutive conserved regions in the human MON1A protein (M2 and M3).(D) Structural superimposition of the coevolution-based 3D model structures for the three MON1A longin repeats. We generated trRosetta “de novo” (without templates) 3D models for human MON1A longin repeats 1, 2, and 3 (Uniprot:Q86VX9 residues: 252–344, 415–518, and 542–641).^23^ The confidence of the predicted models was considered to be very high by trRosetta, with TM-scores of 0.81, 0.85, and 0.78, for repeats 1, 2, and 3, respectively. Structural superimposition was performed using DALI.^57^ Predicted 3D models of MON1A longin repeats 1, 2, and 3 are coloured in red, yellow, and violet, respectively. Structures are presented using Pymol (http://www.pymol.org/). (E) Structural comparison of among the coevolution-based 3D model structures for the three MON1A longin repeats, and the the experimentally determined structure of the first longin repeat of *C. thermophilum* MON1 (PDB: 5LDD_A).^53^ The heat-map is labelled with highly statistically significant DALI Z-scores (above the diagonal) and FATCAT P-values (below the diagonal). The heat-map was generated with DALI (“--matrix” command option).^57^ Numerical values used to assign colour below the diagonal correspond to the negative log10 value of FATCAT P-values.^58^ DALI and FATCAT offer consistent results, showing high degrees of structural similarity among the MON1A longin repeats' models and with the experimentally determined structure of the first longin repeat of *C. thermophilum* MON1 (PDB: 5LDD_A).

Domain limits in protein sequences of unknown structure once could only be predicted by exploiting evolutionary conservation or amino acid compositional biases in linkers and domain boundaries.^47^, ^48^ Prediction accuracy of these methods, however, was typically low. Now, contact and distance prediction maps can reveal well-defined, contiguous features whose sequence likely folds into a well-defined structural domain (Figure 3). New coevolution-based contact and distance prediction methods now allow domains to be identified automatically by methods similar to those used in structural biology, consisting of the recognition of regions with a high density of internal contacts forming compact hydrophobic cores.^44^, ^49^, ^50^, ^51^

We illustrate this approach first with a contact prediction map of human MON1A (Figure 3). Distant homology among MON1A’s three domains (M1-3) was identified, in part, using such a map.^52^ More specifically, its map revealed a three-times-repeated contact pattern in MON1A sequence (indicated by dashed rectangles in Figure 3). Each pattern contained features that project away from the diagonal, indicative of physical contacts between nearby, antiparallel secondary structures. As expected from their continuous stretches of hydrogen bonds, pairs of β-strands are more easily recognizable than interaction patterns involving α-helices.^19^ Figure 3(C) (right, top) shows a contact map calculated from the known tertiary structure of the first longin domain (labelled “M1”) of MON1 from the fungus *Chaetomium thermophilum*.^53^ Note that each axis represents this domain’s sequence and is annotated with the known secondary structures (arrows [β-strands] and cylinders [α-helices]). Features that follow the diagonal are close contacts between near-adjacent residues along the sequence. Features that stick out perpendicular to the diagonal represent contacts between successive anti-parallel β-strands (e.g. strands 1 and 2). By contrast, those features lying away from the diagonal reflect interactions between β-strands that are not adjacent in sequence (i.e. strands 1 and 5). The order of strands within β-sheets can be inferred from contact features present or absent between β-strand pairs.

To infer that these three domains M1-3 were homologous, rather than merely structures adopting the same fold, more conventional sequence database-searching approaches (including HHpred) were then necessary. In this example, sequence divergence among M1-3 domains was so substantial that only by aggregating information from five MON1A paralogues – each with the same three-domain architecture – was distant homology able to be proven.^52^ This example illustrates how structural constraints (obtained via co-evolutionary information) and sequence similarity methods (that fail to consider co-evolutionary patterns) provide complementary information.

### Structural comparison between coevolution-based models and experimentally determined structures.

The substantial improvements provided by coevolution-based methods now permit predicted models of protein structure to be analysed as if they were experimentally determined structures. Structural similarity, when identified among coevolution-based models of different families or with proteins of known structure, can then be investigated as to whether it reflects homology or else analogy. Analogy, resulting from convergent evolution, has occurred often because of the finite number of possible protein folds.^54^, ^55^, ^56^

Conversely, inferred homology based on sequence comparisons can be validated should they be consistent with their structures predicted using co-evolution information. For example, for the case of MON1A whose analysis was presented above, sequence similarity among the different MON1A longin repeats was very modest (with sequence identities below 10%). Nevertheless, co-evolution based predicted structures were highly and statistically similar (Figure 3 panels (D) and (E)),^23^, ^57^, ^58^ thus confirming the previously described homologous relationships based only on sequence similarity.^52^

### Internal repeats

Repeated patterns of sequence conservation facilitate identification of internal duplications in proteins.^59^ Many internal repeats, however, are poorly conserved and such high divergence can preclude their identification. Our example of three highly-divergent domains in MON1A (Figure 3) provide one such instance.

**Figure 4:**
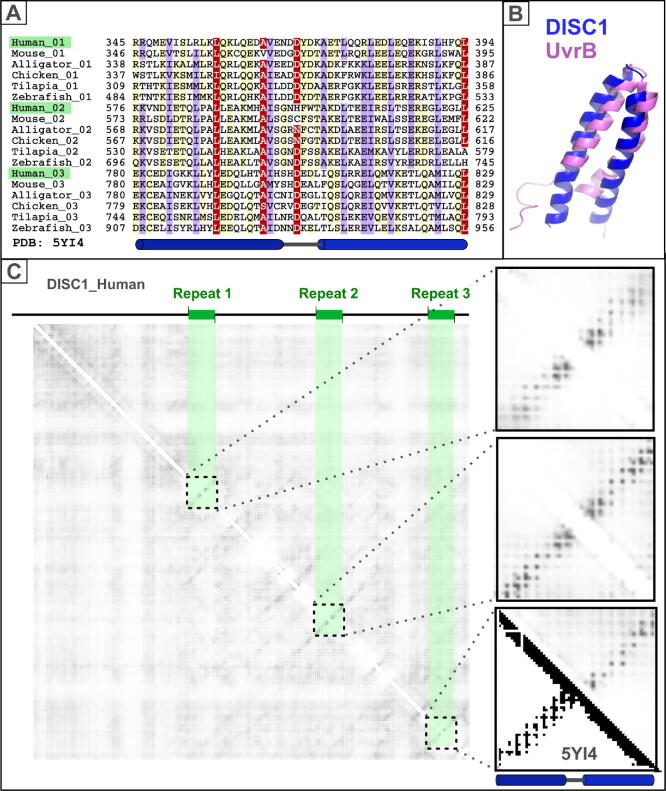
Small UvrB-like repeats in DISC1. (A) Representative multiple sequence alignment of DISC1 UVR-like repeats in vertebrates.^60^ The three repeats in human DISC1 are highlighted in green. The MSA colouring scheme indicates the average BLOSUM62 score (which is correlated with amino acid conservation) for each alignment column: red (scores > 2.5), violet (between 2.5 and 1) and light yellow (between 1 and 0.3). The alignment was generated with T-Coffee^100^ and presented using the program Belvu.^93^ Domain limits are indicated by flanking amino acid numbers. The known secondary structure from *Mus musculus* DISC1 UVR-like third repeat is shown below the alignment (PDB: 5YI4_A).^62^ Alpha-helices are indicated by blue cylinders. Sequences have been named according to their species’ common name. (B) Structural superposition of the *Mus musculus* DISC1 UVR-like third repeat (blue ribbon, PDB: 5YI4_A)^62^ and the *Escherichia coli* UvrB domain (magenta ribbon, PDB: 1E52).^111^ Cartoons were generated using PyMOL (https://pymol.org/). (C) Top: Domain architecture of human DISC1 protein with three UVR-like repeats identified in its sequence indicated by green rectangles and labelled (Repeat 1 to 3).^60^ Left: RaptorX coevolution-based contact prediction, obtained using default parameters, for the human DISC1 protein (amino acids 61 to 854).^22^ A triplicated contact pattern is observed in agreement with our previously proposed UVR-like repeats. Right: Magnified contact map regions corresponding to each UVR-like repeat. Bottom: Comparison of contact maps for the predicted (above diagonal) and actual (below diagonal) maps for the third UVR-like repeat of *Mus musculus* DISC1 (PDB: 5YI4_A).^62^

Repeats can be even more challenging to discern if they are short and/or dispersed throughout the sequence, rather than being joined together in tandem arrays. A once difficult illustration of this is DISC1 (disrupted in schizophrenia 1) whose unusual amino acid composition and high α-helical content obscured three short repeats dispersed across its sequence (Figure 4). A decade ago, detailed sequence analysis revealed each repeat to be a UVR-like pair of antiparallel α-helices.^60^ Nevertheless, perhaps owing to their small size and modest sequence similarity, the validity of these predicted repeats has long been overlooked.^61^, ^62^ With the advent of contact and distance prediction methods,^22^ the three UVR-like repeats become evident. Each repeat’s pair of tightly interacting anti-parallel α-helices is consistent with its associated feature protruding at right-angles out from the diagonal (Figure 4(C)). Comparison of their sequences (Figure 4(A)) demonstrates these repeats’ homology.

### An analogy

Archaeologists, when confronted with a featureless landscape, often employ ground-penetrating radar to identify buried structures.^63^ Signals from this high-resolution and non-invasive technique, once filtered, can show concealed objects as parallel or perpendicular features in a radar map. Without such an efficient technique, archaeologists would continually prospect for high value structures in the wrong locations.

We use this by way of analogy to explain why we believe co-evolution-based contact and structure prediction will be transformative for remote homology inference of domains and repeats. Just as ground-penetrating radar can be applied to many locations, contact maps can be predicted for most sequences. In both approaches, the signal has to be processed and de-noised, and structures once revealed then compared with better-known examples elsewhere. In sum, the greatest value stems from how contact prediction, as with ground-penetrating radar, efficiently divines where structures are located within an otherwise nondescript environment.

### Insertions and alternative conformations

The expectation that domains in proteins are arranged as “beads-on-a-string” is largely supported by known protein structures.^15^ Nevertheless, evolution always throws up exceptions to more general rules, including proteins in which one domain occurs as an insertion within another.^16^, ^17^ Such exceptions have always presented challenges because alignment algorithms typically assume the collinearity and close proximity of aligned sequence segments.

**Figure 5:**
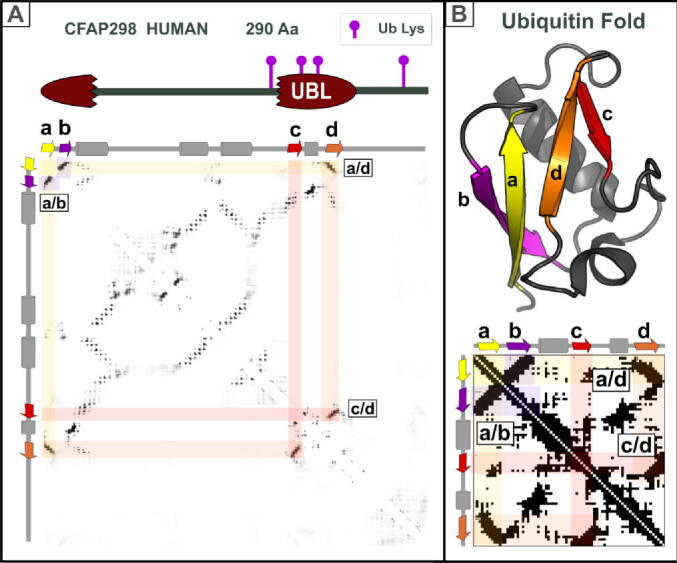
Identification of domain insertions. A new Ubiquitin-like (UBL) domain in CFAP298. (A) Top: Domain architecture of human CFAP298 protein. The broken red oval represents a ubiquitin-like domain containing a long insertion. Ubiquitinated lysines found in high-throughput analysis are indicated with purple pins.^112^, ^113^ Bottom: Contact prediction map for the full-length CFAP298 human protein sequence. PsiPred predicted secondary structures^103^, ^104^ for CFAP298 are shown above the contact map. In Pfam a domain of unknown function (PF11069) is located from position 189 to 285 in UniProt:CF298_HUMAN.^114^ (B) Top: Schematic of the structure of human Ubiquitin-like protein ISG15 (PDB: 2HJ8; amino acids 79 to 153), β-strands are labelled a-to-d and coloured in yellow, purple, red, and orange, respectively. Image was generated using PyMOL (https://pymol.org/). Bottom: Actual contact map calculated from this known Ubiquitin-like domain structure generated using the Cocomaps server (input: 2HJ85_A *versus* 2HJ8_A, cut-off distance value = 7 Å).^110^ Anti-parallel “a/b” and “c/d”, and parallel “a/d”, β-strand pairs within the Ubiquitin fold core, are labelled accordingly.

A contact prediction map, combined with sequence analysis, can help reveal and resolve such incongruities. CFAP298 (also known as C21ORF59) plays a role in motile cilia within outer dynein arm assembly.^64^, ^65^, ^66^ Its homology and domain architecture were unclear, except for a predicted coiled-coil and a C-terminal domain of unknown function. A predicted contact map indicated likely interactions between α-helices or β-strands, but yielded no further clues (Figure 5). Sequence analyses, however, suggested partial similarity between a C-terminal region and a ubiquitin-like domain (data not shown). Curiously, this similarity covered only two β-strands (c and d) of the ubiquitin fold but not its N-terminal β-strands (a-and-b). The RaptorX contact map prediction solved this conundrum by revealing a strong feature pairing β-strand-d with a β-strand over 200 amino acids away, close to the protein’s N-terminus.^22^ Combining the coevolution-based contact map – with its features pairing antiparallel and parallel β-strands – and the sequence conservation analysis, revealed CFAP298 to contain a ubiquitin-like domain, within which a largely α-helical sequence has been inserted (Figure 5(A)). Without the contact map, the full extent of the ubiquitin domain would have remained undiscovered.

**Figure 6:**
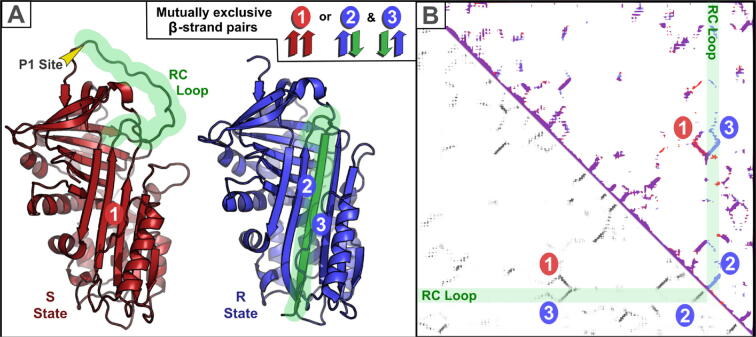
Conformational change in α_1_-antitrypsin. (A) Ribbon diagrams of the two conformational states (Stressed [S] and Relaxed [R] states in red and blue, respectively) of human α_1_-antitrypsin protein (PDB:1QLP and 1EZX).^115^, ^116^ The reactive centre (RC) loop in the S state, which transforms into a long β-strand in the R state, is labelled and coloured in green. The trypsin protease cleavage site is labelled P1. Mutually exclusive β-strand pairs between S and R states are labelled in red (oval, label 1) and blue (oval, label 2), respectively. A parallel pair of β-strands (label 1) in the S state is disrupted by penetration of the RC loop to form two new antiparallel β-strand pairs (ovals, labels 2 and 3). Both diagrams were generated using PyMOL (https://pymol.org/). (B) Above the diagonal is the superimposition of two contact maps obtained from known structures: S and R states of human α_1_-antitrypsin in red and blue, respectively (PDBs:1QLP and 1EZX), generated using the Cocomaps server.^110^ Contacts present in both structures are shown in purple and specific contacts in S or R states are in red or blue, respectively. The green transparent rectangles identify the location of the RC Loop (which transforms into a long β-strand in the R state). Transitioning between the two states, a parallel pair of β-strands in the S state (red oval, label 1) disappears and two antiparallel β-strand pairs appear (blue ovals, labels 2 and 3). Below the diagonal is the RaptorX contact map.^22^ This captures co-evolutionary signals reflecting each of the two (S and R) conformations of α_1_-antitrypsin.

Folded proteins can adopt multiple conformations, for example following proteolytic cleavage of a pro-protein. When first determined, the structure of cleaved α_1_-antitrypsin, a protease inhibitor, revealed that the two ends of its cleaved peptide bond were an astonishing 65 Å apart, implying a large conformational change following proteolytic cleavage.^67^ This stressed-to-relaxed (S → R) transition involves an opening of a β-sheet to accommodate a new β-strand^68^ (Figure 6(A)). This begged the question whether contact map predictions might capture correlated amino acid substitutions involving this β-sheet in both its uncleaved S and cleaved R conformations. Indeed, different contacts are predicted (Figure 6(B), below the diagonal) to occur in each of the S or R state structures (contacts observed in known structures above the diagonal; red and blue respectively). Correlated mutations clearly have occurred that optimise β-strand interactions in two very different structural states. Coevolution-based predicted contacts can thus inform on protein dynamics and conformational change.^69^, ^70^, ^71^, ^72^ This is similar to how comparison of a protein's contact maps calculated from its known structures can reveal its conformational flexibility and dynamics.^73^

## Limits to the New Homology Detection Horizon

Although this new combination of contact map predictions and sequence analysis tools lengthens the horizon of detectable homology, what limitations hinder us from pushing it back yet further? First, protein sequences are imperfectly known. For example the protein-coding status of a substantial minority of human genes remains in question.^74^, ^75^ Other proteins evolve so rapidly that MSAs cannot be accurately constructed across diverse phyla. For such proteins, contact maps will be less able to be predicted accurately. Deep mutagenesis experiments generating diverse artificial sequences through in vitro evolution may overcome this limitation.^34^, ^35^

Second, current methods are less accurate when predicting contacts between secondary structures that are well separated in amino acid sequence. Predicted contact maps for large domains, or domains with large insertions, will thus tend to be less accurate. Third, protein folds, and their internal contacts, are not immutable. Rather, they can adopt multiple conformations (as for α_1_-antitrypsin, above) or over evolution convert from one fold to another by insertions, deletions, circular permutations, strand swaps and secondary structure transformations.^16^, ^17^, ^76^, ^77^, ^78^, ^79^, ^80^, ^81^ Such topological changes may partly obscure evidence for distant homology arising from contact map predictions.

Other limitations relate to how an evolutionary observation (homology) is interpreted functionally. Inferring protein function from homology is complex, particularly at great evolutionary distances,^82^ largely because function itself is not well-defined.^83^ A protein’s function can be approximated by its molecular interactions,^84^ so accurate predictions of contacts between interacting proteins would greatly assist functional inference. Nevertheless, current coevolution-based contact prediction methods cannot predict contacts in protein binding sites accurately. They also are unable to distinguish amino acid contacts in monomers *versus* homo-oligomeric structures.

## Five Outstanding Questions

Co-evolution-based protein structure prediction, when combined with traditional sequence-based homology inference, are now further extending the detection horizon of homology. The locations and structures of domains and repeats can be unearthed with an impact in evolutionary structural biology just as profound as ground-penetrating radar in archaeology. Advances in this area have been rapid, and answers to the following questions may be rapidly forthcoming: (1) Nearly half of all amino acid residues fall outside of a sequence family annotations.^85^ Can these methods begin to illuminate this large fraction of the “dark proteome” whose structure is currently unknown.^86^, ^87^ (2) How many new folds and domain or repeat families will become identifiable? (3) Will these more comprehensive predictions be completely automated and their results served to the wider community as data resources? (4) Can structure and function be revealed that are specific to particular evolutionary lineages? (5) Can protein interaction prediction methods be devised that permit functional mechanisms to be inferred?.^88^, ^89^, ^90^

## Conclusions

Experimentally determined protein structures have frequently revealed unexpected relationships between protein families.^91^, ^92^ Similarly, remote homology identification between domains can now be enhanced by comparing coevolution-based structure predictions and experimentally characterised protein structures. The new coevolution-based protein structural models now promise to further extend the homology horizon, and unite predictions of protein structure and evolution.

## CRediT authorship contribution statement

**Luis Sanchez-Pulido:** Data curation, Writing - original draft. **Chris P. Ponting:** Data curation, Writing - original draft.

## References

[1] Loewenstein Y., Raimondo D., Redfern O.C., Watson J., Frishman D., Linial M., Orengo C., Thornton J. (2009). Protein function annotation by homology-based inference. Genome Biol..

[2] Pearson W.R., Sierk M.L. (2005). The limits of protein sequence comparison?. Curr. Opin. Struct. Biol..

[3] Ponting C.P., Schultz J., Copley R.R., Andrade M.A., Bork P. (2000). Evolution of domain families. Adv. Protein Chem..

[4] Rost B. (1999). Twilight zone of protein sequence alignments. Protein Eng..

[5] Altschul S.F., Madden T.L., Schäffer A.A., Zhang J., Zhang Z., Miller W., Lipman D.J. (1997). Gapped BLAST and PSI-BLAST: A new generation of protein database search programs. Nucleic Acids Res..

[6] Finn R.D., Clements J., Arndt W., Miller B.L., Wheeler T.J., Schreiber F., Bateman A., Eddy S.R. (2015). HMMER web server: 2015 Update. Nucleic Acids Res..

[7] Koonin E.V., Wolf Y.I., Aravind L. (2000). Protein fold recognition using sequence profiles and its application in structural genomics. Adv. Protein Chem..

[8] Söding J. (2005). Protein homology detection by HMM-HMM comparison. Bioinformatics.

[9] Zimmermann L., Stephens A., Nam S.Z., Rau D., Kübler J., Lozajic M., Gabler F., Söding J., Lupas A.N., Alva V. (2018). A completely reimplemented MPI bioinformatics toolkit with a new HHpred server at its core. J. Mol. Biol..

[10] Sanchez-Pulido L., Diffley J.F.X., Ponting C.P. (2010). Homology explains the functional similarities of Treslin/Ticrr and Sld3. Curr. Biol..

[11] Ponting C.P., Russell R.R. (2002). The natural history of protein domains. Annu. Rev. Biophys. Biomol. Struct..

[12] Todd A.E., Orengo C.A., Thornton J.M. (2001). Evolution of function in protein superfamilies, from a structural perspective. J. Mol. Biol..

[13] Vogel C., Berzuini C., Bashton M., Gough J., Teichmann S.A. (2004). Supra-domains: evolutionary units larger than single protein domains. J. Mol. Biol..

[14] Paladin L., Bevilacqua M., Errigo S., Piovesan D., Mičetić I., Necci M., Monzon A.M., Fabre M.L., Lopez J.L., Nilsson J.F., Rios J., Menna P.L., Cabrera M., Buitron M.G., Kulik M.G., Fernandez-Alberti S., Fornasari M.S., Parisi G., Lagares A., Hirsh L., Andrade-Navarro M.A., Kajava A.V., Tosatto S.C.E. (2021). RepeatsDB in 2021: Improved data and extended classification for protein tandem repeat structures. Nucleic Acids Res..

[15] Heringa J., Taylor W.R. (1997). Three-dimensional domain duplication, swapping and stealing. Curr. Opin. Struct. Biol..

[16] Russell R.B., Ponting C.P. (1998). Protein fold irregularities that hinder sequence analysis. Curr. Opin. Struct. Biol..

[17] Grishin N.V. (2001). Fold change in evolution of protein structures. J. Struct. Biol..

[18] D’Alfonso G., Tramontano A., Lahm A. (2001). Structural conservation in single-domain proteins: implications for homology modeling. J. Struct. Biol..

[19] Godzik A., Skolnick J., Kolinski A. (1993). Regularities in interaction patterns of globular proteins. Protein Eng. Des. Sel..

[20] Holm L., Sander C. (1993). Protein structure comparison by alignment of distance matrices. J. Mol. Biol..

[21] Xu J. (2019). Distance-based protein folding powered by deep learning. Proc. Natl. Acad. Sci. USA.

[22] Wang S., Sun S., Li Z., Zhang R., Xu J. (2017). Accurate de novo prediction of protein contact map by ultra-deep learning model. PLoS Comput. Biol..

[23] Yang J., Anishchenko I., Park H., Peng Z., Ovchinnikov S., Baker D. (2020). Improved protein structure prediction using predicted interresidue orientations. Proc. Natl. Acad. Sci. USA.

[24] Norn C., Wicky B.I.M., Juergens D., Liu S., Kim D., Tischer D., Koepnick B., Anishchenko I., Players F., Baker D., Ovchinnikov S. (2021). Protein sequence design by conformational landscape optimization. Proc. Natl. Acad. Sci. USA.

[25] Senior A.W., Evans R., Jumper J., Kirkpatrick J., Sifre L., Green T., Qin C., Žídek A., Nelson A.W.R., Bridgland A., Penedones H., Petersen S., Simonyan K., Crossan S., Kohli P., Jones D.T., Silver D., Kavukcuoglu K., Hassabis D. (2020). Improved protein structure prediction using potentials from deep learning. Nature.

[26] Callaway E. (2020). “It will change everything”: DeepMind’s AI makes gigantic leap in solving protein structures. Nature.

[27] Breen M.S., Kemena C., Vlasov P.K., Notredame C., Kondrashov F.A. (2012). Epistasis as the primary factor in molecular evolution. Nature.

[28] Göbel U., Sander C., Schneider R., Valencia A. (1994). Correlated mutations and residue contacts in proteins. Proteins Struct. Funct. Bioinforma..

[29] Shindyalov I.N., Kolchanov N.A., Sander C. (1994). Can three-dimensional contacts in protein structures be predicted by analysis of correlated mutations?. Protein Eng. Des. Sel..

[30] Altschuh D., Lesk A.M., Bloomer A.C., Klug A. (1987). Correlation of co-ordinated amino acid substitutions with function in viruses related to tobacco mosaic virus. J. Mol. Biol..

[31] Lim W.A., Sauer R.T. (1989). Alternative packing arrangements in the hydrophobic core of λrepresser. Nature.

[32] Camps M., Herman A., Loh E., Loeb L.A. (2007). Genetic constraints on protein evolution. Crit. Rev. Biochem. Mol. Biol..

[33] Marks D.S., Colwell L.J., Sheridan R., Hopf T.A., Pagnani A., Zecchina R., Sander C. (2011). Protein 3D structure computed from evolutionary sequence variation. PLoS One.

[34] Rollins N.J., Brock K.P., Poelwijk F.J., Stiffler M.A., Gauthier N.P., Sander C., Marks D.S. (2019). Inferring protein 3D structure from deep mutation scans. Nature Genet..

[35] Schmiedel J.M., Lehner B. (2019). Determining protein structures using deep mutagenesis. Nature Genet..

[36] Sathyapriya R., Duarte J.M., Stehr H., Filippis I., Lappe M. (2009). Defining an essence of structure determining residue contacts in proteins. PLoS Comput. Biol..

[37] Jones D.T., Buchan D.W.A., Cozzetto D., Pontil M. (2012). PSICOV: Precise structural contact prediction using sparse inverse covariance estimation on large multiple sequence alignments. Bioinformatics.

[38] Kamisetty H., Ovchinnikov S., Baker D. (2013). Assessing the utility of coevolution-based residue-residue contact predictions in a sequence- and structure-rich era. Proc. Natl. Acad. Sci. USA.

[39] Hopf T.A., Colwell L.J., Sheridan R., Rost B., Sander C., Marks D.S. (2012). Three-dimensional structures of membrane proteins from genomic sequencing. Cell.

[40] Hopf T.A., Schärfe C.P.I., Rodrigues J.P.G.L.M., Green A.G., Kohlbacher O., Sander C., Bonvin A.M.J.J., Marks D.S. (2014). Sequence co-evolution gives 3D contacts and structures of protein complexes. Elife.

[41] Li Y., Zhang C., Bell E.W., Zheng W., Zhou X., Yu D.J., Zhang Y. (2020). Deducing high-accuracy protein contact-maps from a triplet of coevolutionary matrices through deep residual convolutional networks. PLoS Comput Biol..

[42] Kandathil S.M., Greener J.G., Jones D.T. (2019). Prediction of interresidue contacts with DeepMetaPSICOV in CASP13. Proteins Struct. Funct. Bioinforma..

[43] Hou, J., Wu, T., Guo, Z., Quadir, F. & Cheng, J. (2020). The MULTICOM protein structure prediction server empowered by deep learning and contact distance prediction. Methods Mol. Biol. 13–26. Doi: 10.1007/978-1-0716-0708-4_2. 10.1007/978-1-0716-0708-4_2 32621217

[44] Zheng W., Zhou X., Wuyun Q., Pearce R., Li Y., Zhang Y. (2020). FUpred: Detecting protein domains through deep-learning-based contact map prediction. Bioinformatics.

[45] Feng S.H., Zhang W.X., Yang J., Yang Y., Bin Shen H. (2020). Topology prediction improvement of α-helical transmembrane proteins through helix-tail modeling and multiscale deep learning fusion. J. Mol. Biol..

[46] Wang S., Fei S., Wang Z., Li Y., Xu J., Zhao F., Gao X. (2019). PredMP: A web server for de novo prediction and visualization of membrane proteins. Bioinformatics.

[47] Liu J., Rost B. (2004). CHOP proteins into structural domain-like fragments. Proteins Struct. Funct. Genet..

[48] Bryson K., Cozzetto D., Jones D. (2007). Computer-assisted protein domain boundary prediction using the dom-pred server. Curr. Protein Pept. Sci..

[49] Holm L., Sander C. (1994). Parser for protein folding units. Proteins Struct. Funct. Bioinforma..

[50] Swindells M.B. (1995). A procedure for detecting structural domains in proteins. Protein Sci..

[51] Siddiqui A.S., Barton G.J. (1995). Continuous and discontinuous domains: an algorithm for the automatic generation of reliable protein domain definitions. Protein Sci..

[52] Sanchez-Pulido L., Ponting C.P. (2020). Hexa-Longin domain scaffolds for inter-Rab signalling. Bioinformatics.

[53] Kiontke S., Langemeyer L., Kuhlee A., Schuback S., Raunser S., Ungermann C., Kümmel D. (2017). Architecture and mechanism of the late endosomal Rab7-like Ypt7 guanine nucleotide exchange factor complex Mon1-Ccz1. Nature Commun..

[54] Russell R.B., Saqi M.A., Sayle R.A., Bates P.A., Sternberg M.J. (1997). Recognition of analogous and homologous protein folds: analysis of sequence and structure conservation. J. Mol. Biol..

[55] Cheng H., Kim B.H., Grishin N.V. (2008). Discrimination between distant homologs and structural analogs: lessons from manually constructed, reliable data sets. J. Mol. Biol..

[56] Krishna S.S., Grishin N.V. (2004). Structurally analogous proteins do exist!. Structure.

[57] Holm L. (2020). DALI and the persistence of protein shape. Protein Sci..

[58] Li Z., Jaroszewski L., Iyer M., Sedova M., Godzik A. (2020). FATCAT 2.0: towards a better understanding of the structural diversity of proteins. Nucleic Acids Res..

[59] Andrade M.A., Perez-Iratxeta C., Ponting C.P. (2001). Protein repeats: structures, functions, and evolution. J. Struct. Biol..

[60] Sanchez-Pulido L., Ponting C.P. (2011). Structure and evolutionary history of DISC1. Hum. Mol. Genet..

[61] Ye F., Kang E., Yu C., Qian X., Jacob F., Yu C., Mao M., Poon R.Y.C. (2017). DISC1 regulates neurogenesis via modulating kinetochore attachment of Ndel1/Nde1 during mitosis. Neuron.

[62] Wang X., Ye F., Wen Z., Guo Z., Yu C., Huang W.K., Rojas Ringeling F., Su Y. (2021). Structural interaction between DISC1 and ATF4 underlying transcriptional and synaptic dysregulation in an iPSC model of mental disorders. Mol. Psychiatry.

[63] Bernardini F., Vinci G., Horvat J., De Min A., Forte E., Furlani S., Lenaz D., Pipan M. (2015). Early Roman military fortifications and the origin of Trieste, Italy. Proc. Natl. Acad. Sci. USA.

[64] Austin-Tse C., Halbritter J., Zariwala M.A., Gilberti R.M., Gee H.Y., Hellman N., Pathak N., Liu Y. (2013). Zebrafish ciliopathy screen plus human mutational analysis identifies C21orf59 and CCDC65 defects as causing primary ciliary dyskinesia. Am. J. Hum. Genet..

[65] Jaffe K.M., Grimes D.T., Schottenfeld-Roames J., Werner M.E., Ku T.S.J., Kim S.K., Pelliccia J.L., Morante N.F.C. (2016). C21orf59/kurly controls both cilia motility and polarization. Cell Rep..

[66] Laura M.H., Yasmine C.B., Raphaël P., Lotfi S., Hugues P.M. (2021). The orthopedic characterization of cfap298 tm304 mutants validate zebrafish to faithfully model human AIS. Sci. Rep..

[67] Loebermann H., Tokuoka R., Deisenhofer J., Huber R. (1984). Human α1-proteinase inhibitor. Crystal structure analysis of two crystal modifications, molecular model and preliminary analysis of the implications for function. J. Mol. Biol..

[68] Stein P., Chothia C. (1991). Serpin tertiary structure transformation. J. Mol. Biol..

[69] Jana B., Morcos F., Onuchic J.N. (2014). From structure to function: the convergence of structure based models and co-evolutionary information. Phys. Chem. Chem. Phys..

[70] Parisi G., Zea D.J., Monzon A.M., Marino-Buslje C. (2015). Conformational diversity and the emergence of sequence signatures during evolution. Curr. Opin. Struct. Biol..

[71] Sutto L., Marsili S., Valencia A., Gervasio F.L. (2015). From residue coevolution to protein conformational ensembles and functional dynamics. Proc. Natl. Acad. Sci. USA.

[72] Sfriso P., Duran-Frigola M., Mosca R., Emperador A., Aloy P., Orozco M. (2016). Residues coevolution guides the systematic identification of alternative functional conformations in proteins. Structure.

[73] Iyer M., Li Z., Jaroszewski L., Sedova M., Godzik A. (2020). Difference contact maps: from what to why in the analysis of the conformational flexibility of proteins. PLoS One.

[74] Abascal F., Juan D., Jungreis I., Martinez L., Rigau M., Rodriguez J.M., Vazquez J., Tress M.L. (2018). Loose ends: almost one in five human genes still have unresolved coding status. Nucleic Acids Res..

[75] Frankish A., Diekhans M., Jungreis I., Lagarde J., Loveland J.E., Mudge J.M., Sisu C., Wright J.C. (2021). GENCODE 2021. Nucleic Acids Res..

[76] Alva V., Koretke K.K., Coles M., Lupas A.N. (2008). Cradle-loop barrels and the concept of metafolds in protein classification by natural descent. Curr. Opin. Struct. Biol..

[77] Andreeva A., Murzin A.G. (2006). Evolution of protein fold in the presence of functional constraints. Curr. Opin. Struct. Biol..

[78] Belogurov G.A., Vassylyeva M.N., Svetlov V., Klyuyev S., Grishin N.V., Vassylyev D.G.G., Artsimovitch I. (2007). Structural basis for converting a general transcription factor into an operon-specific virulence regulator. Mol. Cell..

[79] Gunn A.R., Banos-Pinero B., Paschke P., Sanchez-Pulido L., Ariza A., Day J., Emrich M., Leys D. (2016). The role of ADP-ribosylation in regulating DNA interstrand crosslink repair. J. Cell Sci..

[80] Grishin N.V. (2001). KH domain: one motif, two folds. Nucleic Acids Res..

[81] Liu Y., Eisenberg D. (2002). 3D domain swapping: as domains continue to swap. Protein Sci..

[82] Pearson W.R. (2013). An introduction to sequence similarity (“homology”) searching. Curr. Protoc. Bioinforma.

[83] Bork P. (2000). Powers and pitfalls in sequence analysis: the 70% hurdle. Genome Res..

[84] Bork P., Dandekar T., Diaz-Lazcoz Y., Eisenhaber F., Huynen M., Yuan Y. (1998). Predicting function: from genes to genomes and back. J. Mol. Biol..

[85] Mistry J., Chuguransky S., Williams L., Qureshi M., Salazar G.A., Sonnhammer E.L.L., Tosatto S.C.E., Paladin L. (2021). Pfam: the protein families database in 2021. Nucleic Acids Res..

[86] Perdigão N., Heinrich J., Stolte C., Sabir K.S., Buckley M.J., Tabor B., Signal B., Gloss B.S. (2015). Unexpected features of the dark proteome. Proc. Natl. Acad. Sci. USA.

[87] Wood V., Lock A., Harris M.A., Rutherford K., Bähler J., Oliver S.G. (2019). Hidden in plain sight: What remains to be discovered in the eukaryotic proteome?. Open Biol..

[88] Pazos F., Valencia A. (2002). In silico two-hybrid system for the selection of physically interacting protein pairs. Proteins Struct. Funct. Genet..

[89] Green A.G., Elhabashy H., Brock K.P., Maddamsetti R., Kohlbacher O., Marks D.S. (2021). Large-scale discovery of protein interactions at residue resolution using co-evolution calculated from genomic sequences. Nature Commun..

[90] Jing, X., Zeng, H., Wang, S., & Xu, J., (2020). A web-based protocol for interprotein contact prediction by deep learning. Methods Mol. Biol. 67–80. Doi: 10.1007/978-1-4939-9873-9_6. 10.1007/978-1-4939-9873-9_6 31583631

[91] Fédry J., Liu Y., Péhau-Arnaudet G., Pei J., Li W., Tortorici M.A., Traincard F., Meola A. (2017). The ancient gamete fusogen HAP2 is a eukaryotic class II fusion protein. Cell.

[92] Flaherty K.M., DeLuca-Flaherty C., McKay D.B. (1990). Three-dimensional structure of the ATPase fragment of a 70K heat-shock cognate protein. Nature.

[93] Sonnhammer E.L.L., Hollich V. (2005). Scoredist: A simple and robust protein sequence distance estimator. BMC Bioinformatics.

[94] Beamer L.J., Pabo C.O. (1992). Refined 1.8 Å crystal structure of the λ repressor-operator complex. J. Mol. Biol..

[95] Holm L., Sander C. (1996). Mapping the protein universe. Science.

[96] Burley S.K., Bhikadiya C., Bi C., Bittrich S., Chen L., Crichlow G.V., Christie C.H., Dalenberg K. (2021). RCSB Protein Data Bank: powerful new tools for exploring 3D structures of biological macromolecules for basic and applied research and education in fundamental biology, biomedicine, biotechnology, bioengineering and energy sciences. Nucleic Acids Res..

[97] Suzek B.E., Wang Y., Huang H., McGarvey P.B., Wu C.H. (2015). UniRef clusters: A comprehensive and scalable alternative for improving sequence similarity searches. Bioinformatics.

[98] Letunic I., Khedkar S., Bork P. (2021). SMART: recent updates, new developments and status in 2020. Nucleic Acids Res..

[99] Blum M., Chang H.Y., Chuguransky S., Grego T., Kandasaamy S., Mitchell A., Nuka G., Paysan-Lafosse T. (2021). The InterPro protein families and domains database: 20 years on. Nucleic Acids Res..

[100] Garriga E., Di Tommaso P., Magis C., Erb I., Mansouri L., Baltzis A., Floden E., Notredame C. (2021). Multiple sequence alignment computation using the T-coffee regressive algorithm implementation. Methods Mol. Biol..

[101] Lupas A., Van Dyke M., Stock J. (1991). Predicting coiled coils from protein sequences. Science.

[102] Krogh A., Larsson B., Von Heijne G., Sonnhammer E.L.L. (2001). Predicting transmembrane protein topology with a hidden Markov model: application to complete genomes. J. Mol. Biol..

[103] Buchan D.W.A., Jones D.T. (2019). The PSIPRED protein analysis workbench: 20 years on. Nucleic Acids Res..

[104] Jones D.T. (1999). Protein secondary structure prediction based on position-specific scoring matrices. J. Mol. Biol..

[105] Wang Y., Zhang H., Zhong H., Xue Z. (2021). Protein domain identification methods and online resources. Comput. Struct. Biotechnol. J..

[106] Sillitoe I., Dawson N., Thornton J., Orengo C. (2015). The history of the CATH structural classification of protein domains. Biochimie.

[107] Todd A.E., Marsden R.L., Thornton J.M., Orengo C.A. (2005). Progress of structural genomics initiatives: an analysis of solved target structures. J. Mol. Biol..

[108] Szklarczyk D., Gable A.L., Nastou K.C., Lyon D., Kirsch R., Pyysalo S., Doncheva N.T., Legeay M. (2021). The STRING database in 2021: customizable protein-protein networks, and functional characterization of user-uploaded gene/measurement sets. Nucleic Acids Res..

[109] Kinch L.N., Grishin N.V. (2006). Longin-like folds identified in CHiPS and DUF254 proteins: Vesicle trafficking complexes conserved in eukaryotic evolution. Protein Sci..

[110] Vangone A., Spinelli R., Scarano V., Cavallo L., Oliva R. (2011). COCOMAPS: A web application to analyze and visualize contacts at the interface of biomolecular complexes. Bioinformatics.

[111] Alexandrovich A., Czisch M., Frenkiel T.A., Kelly G.P., Goosen N., Moolenaar G.F., Chowdhry B.Z., Sanderson M.R. (2001). Solution structure, hydrodynamics and thermodynamics of the UvrB C-terminal domain. J. Biomol. Struct. Dyn..

[112] Denis N.J., Vasilescu J., Lambert J.P., Smith J.C., Figeys D. (2007). Tryptic digestion of ubiquitin standards reveals an improved strategy for identifying ubiquitinated proteins by mass spectrometry. Proteomics.

[113] Kim W., Bennett E.J., Huttlin E.L., Guo A., Li J., Possemato A., Sowa M.E., Rad R. (2011). Systematic and quantitative assessment of the ubiquitin-modified proteome. Mol. Cell..

[114] Bateman A., Coggill P., Finn R.D. (2010). DUFs: families in search of function. Acta Crystallogr. Sect. F Struct. Biol. Cryst. Commun..

[115] Elliott P.R., Pei X.Y., Dafforn T.R., Lomas D.A. (2000). Topography of a 2.0 Å structure of α 1 -antitrypsin reveals targets for rational drug design to prevent conformational disease. Protein Sci..

[116] Huntington J.A., Read R.J., Carrell R.W. (2000). Structure of a serpin-protease complex shows inhibition by deformation. Nature.

